# Successful anticoagulant therapy for disseminated intravascular coagulation during conservative management of placenta percreta: a case report and literature review

**DOI:** 10.1186/s12884-017-1634-8

**Published:** 2017-12-29

**Authors:** Shinya Matsuzaki, Kiyoshi Yoshino, Masayuki Endo, Takuji Tomimatsu, Tsuyoshi Takiuchi, Kazuya Mimura, Keiichi Kumasawa, Yutaka Ueda, Tadashi Kimura

**Affiliations:** Department of Obstetrics and Gynecology, Osaka University Graduate School of Medicine, 2-2 Yamadaoka, Suita, Osaka, 565-0871 Japan

**Keywords:** Placenta percreta, Conservative management, Disseminated intravascular coagulation, Fever, Transverse uterine fundal incision

## Abstract

**Background:**

Placenta percreta is a rare obstetric condition associated with the risk of massive intraoperative hemorrhage. Recently, conservative management of placenta percreta has been performed to reduce maternal morbidity. However, various complications have been reported during such management. Only a few cases of asymptomatic disseminated intravascular coagulation (DIC) or fever without infection have been reported. Here, we discuss such a case and review the related literature to understand this rare condition better. For this, we performed an electronic literature review.

**Case presentation:**

We present the clinical course, results of blood tests, and serial magnetic resonance images of a 35-year-old female (gravida 5, para 2) with placenta percreta complicated by placenta previa that was managed conservatively. The patient successfully delivered a healthy baby by a cesarean delivery via a transverse uterine fundal incision at 36 weeks of gestation. We did not observe intraoperative complications during cesarean delivery, and the postoperative course remained uncomplicated until 47 days after the delivery. However, asymptomatic DIC developed after 47 days, and her serum fibrinogen level declined to 42 mg/dL, which was successfully treated with anticoagulant therapy by a therapeutic dose of intravenous heparin for 22 days (postoperative days 48–69). Although DIC resolved, subsequent fever persisted for approximately 1 month (postoperative days 67–103). Infection was ruled out, and conservative management was successfully continued.

Literature review revealed that successful conservative management of a patient with asymptomatic DIC and subsequent fever without infection is extremely rare.

**Conclusions:**

Some patients with DIC and fever can continue conservative management of placenta percreta, although careful examination and monitoring are needed.

## Background

Placent accreta is one of the most common cause of postpartum hemorrhage [1–3] and placenta percreta is the most severe variant of placenta accreta in which the placenta abnormally penetrates the myometrium [4]. It is associated with heavy obstetrical hemorrhage and bladder injury [5–7]. The average blood loss during surgery for placenta percreta is 4800 mL [5]; such heavy hemorrhage causes high morbidity and complicates the procedure [8, 9]. The standard treatment for placenta accreta, including placenta percreta, is cesarean hysterectomy [10–12]; however, some surgeons choose conservative management to avoid potential intraoperative complications [8]. Nevertheless, complications of placenta percreta have been reported with conservative management, including sepsis, disseminated intravascular coagulation (DIC), massive hemorrhage, and uterine infection [13, 14].

We report on a patient with this rare complication during conservative management of placenta percreta. Additionally, we discuss the current literature on this topic.

## Case presentation

**Fig. 1:**
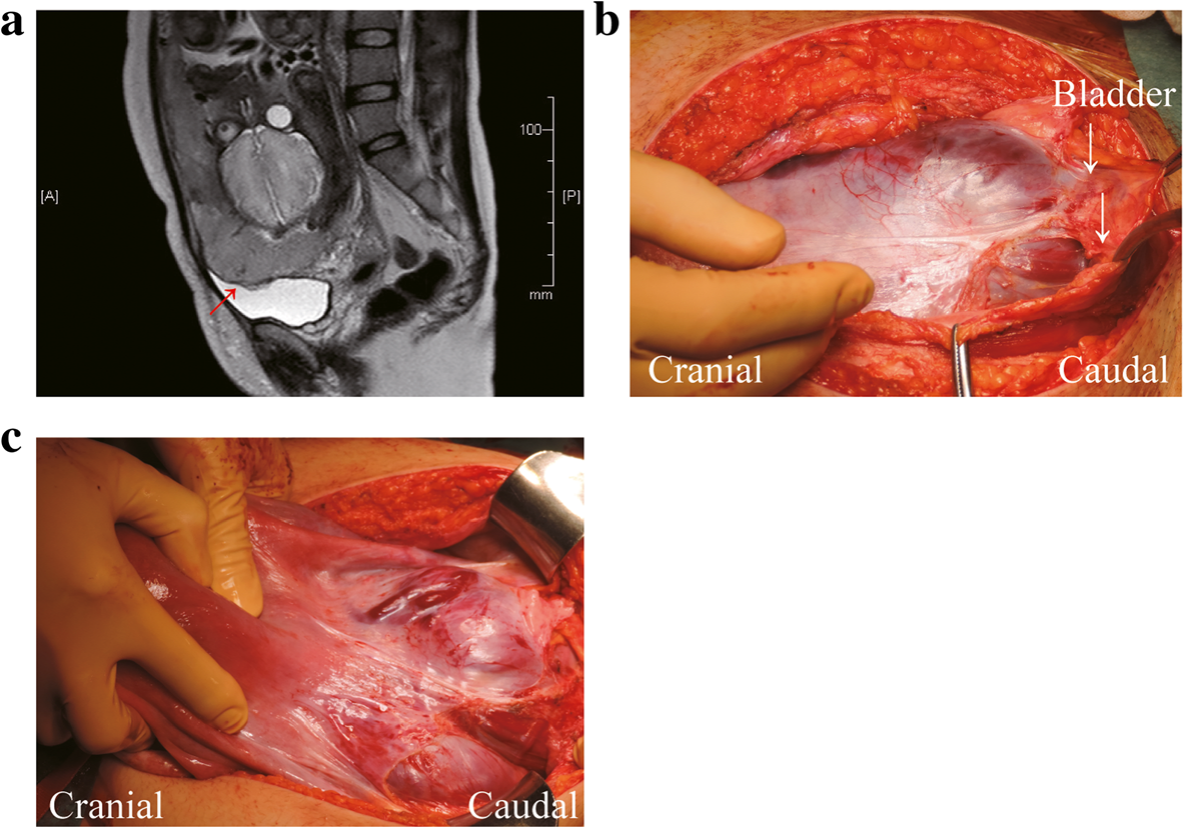
**a** A pelvic MRI was performed at 35 weeks of gestation. Red arrow indicates the loss of uterine myometrium between the placenta and the bladder wall. Based these findings, we suspected placenta percreta with bladder involvement. **b** and **c**, intraoperative findings revealed that placenta percreta involved the bladder and approached the pelvic sidewall and filled the cul de sac. White arrow indicates strong adhesion between the placenta and bladder

A 35-year-old woman (gravida 5, para 2) was referred to our hospital due to placenta previa at 34 weeks of gestation. Her previous pregnancies involved two cesarean deliveries, one spontaneous abortion and one induced abortion. She had no medical history, and during her current pregnancy, the placenta covered the entire anterior wall of the lower uterine segment, and she was diagnosed with placenta previa marginalis. Ultrasonographic findings revealed loss of a clear zone between the placenta and myometrium. Magnetic resonance imaging (MRI) revealed loss of the uterine myometrium between the placenta and bladder walls and broad adhesion between the uterus and bladder (Fig. 1a). Based on these findings, the patient was considered to be at high risk for placenta percreta. Fetal growth was appropriate for the gestational age. The patient was counseled regarding the complications associated with cesarean hysterectomy and the conservative management of placenta percreta, and we informed her about the severity of complications associated with the failure of conservative management. The patient expressed a desire to undergo conservative management to avoid potential intraoperative complications and not to preserve fertility.

A planned cesarean delivery was performed at 36 weeks of gestation. Laparotomy, which was started under combined spinal–epidural anesthesia, revealed large blood vessels and the placenta penetrating through the anterior uterine wall and strong and broad adhesion between placenta and bladder wall was observed (Fig. 1b). Therefore, we suspected placenta percreta and determined that separating the bladder from the uterus would be extremely difficult. We made a transverse uterine fundal incision to avoid an incision into the placenta; a healthy male infant weighing 2312 g was delivered successfully. A uterine fundal incision demonstrated minimal bleeding from the incision site, and could avoid the iatrogenic partial separation of the placenta. After cesarean delivery, the uterus was well contracted, and no bleeding was observed from the placental site (Fig. 1c).

A multidisciplinary team comprising obstetrics, perinatology, gynecologic oncology, and urology diagnosed her with placenta percreta with bladder involvement, approaching the pelvic sidewall and filling the cul de sac. Accordingly, we decided on conservative management to minimize potential surgical morbidity. The intraoperative blood loss estimated by the weight of the swab was 200 mL. The patient was not administered postoperative prophylactic antibiotics. She had an uncomplicated intra- and postoperative course and was discharged on postoperative day 14 with her infant in good condition. The patient was followed up at weekly intervals and examined for general condition determined by blood tests and ultrasonography.

**Fig. 2:**
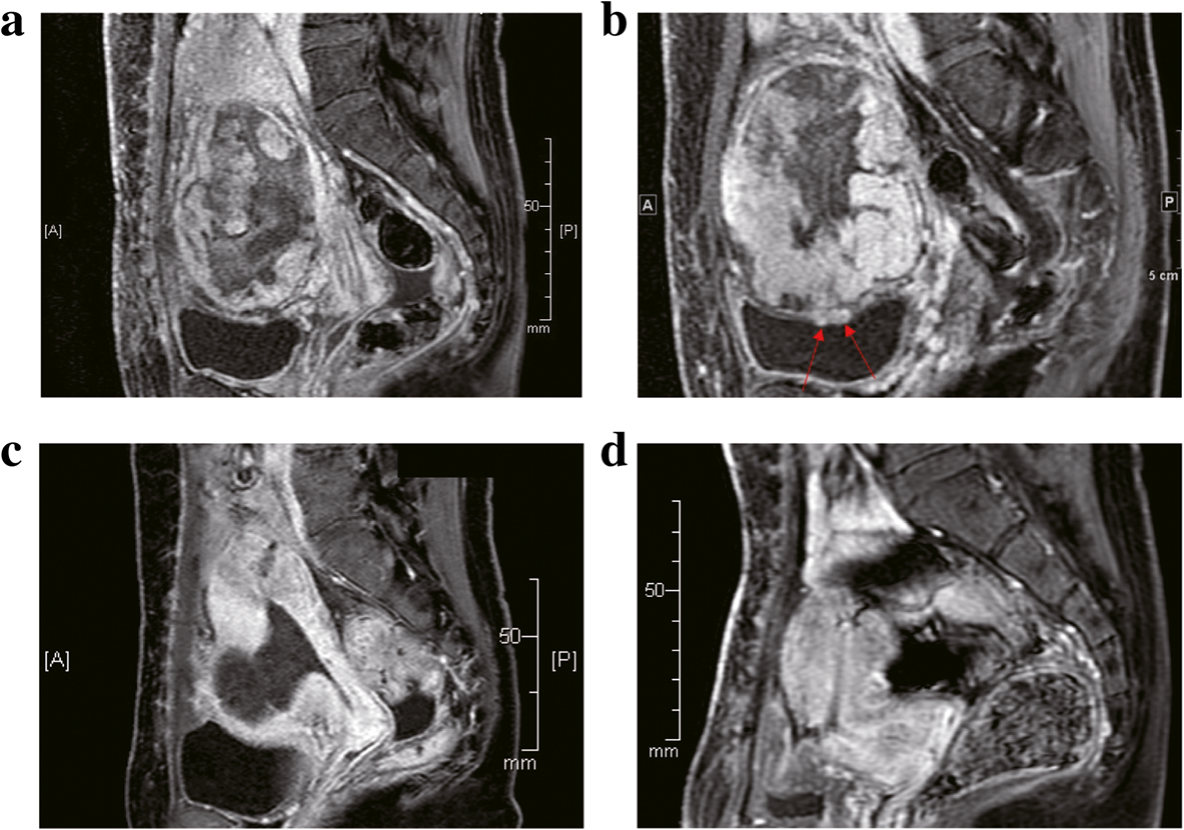
A pelvic MRI was performed on postoperative days 48 (**a** and **b**), 92 (**c**), and 232 (**d**). **a** and **b**, MRI on postoperative day 48 revealed a decrease in the size of the placenta (approximately 12 cm). Gadolinium-enhanced T1-weighted MRI revealed a diffuse lesion with variable enhancement of the placenta. The other slice of the image is shown in **b**. Red arrows in (**b**), the residual placenta that had invaded the bladder wall. **c**, MRI on postoperative day 92 revealed a further decrease in the size of the placenta (approximately 6 cm). Gadolinium-enhanced T1-weighted MRI exhibited no enhancement of the placenta. **d**, MRI on postoperative day 232 confirmed the absence of any residual placenta. With gadolinium enhancement, a small defect was observed in the uterine anterior wall

Her clinical course was unremarkable until postoperative day 42 when blood tests revealed that her serum fibrinogen level had decreased to 114 mg/dL (normal range, 150–350 mg/dL). By day 46, her serum fibrinogen level had decreased further to 62 mg/dL. On day 47, she was admitted to our hospital. Significant coagulation abnormalities were observed in her blood laboratory parameters: serum fibrinogen level, 42 mg/dL; activated partial thromboplastin time, 37 s (normal, 24–39 s); prothrombin time, 59% (normal, 70.0%–125.0%); and D-dimer level, 40.35 μg/mL (normal, <0.5 μg/mL). These results suggested the onset of DIC. The results of other blood tests are shown in Table 1. A careful physical examination and blood tests showed no sign of infection or bleeding; therefore, we considered that DIC likely was caused by the residual placenta. MRI performed on postoperative day 48 revealed that the placenta remained and that its vasculature extended to the bladder wall (Fig. 2a and b). Although we considered delayed hysterectomy as high risk, continuing the conservative management posed an even higher risk. However, the patient strongly insisted on continuing conservative management despite being informed about the associated risks. Therefore, we decided to continue with conservative management instead of performing hysterectomy.

**Table 1:**
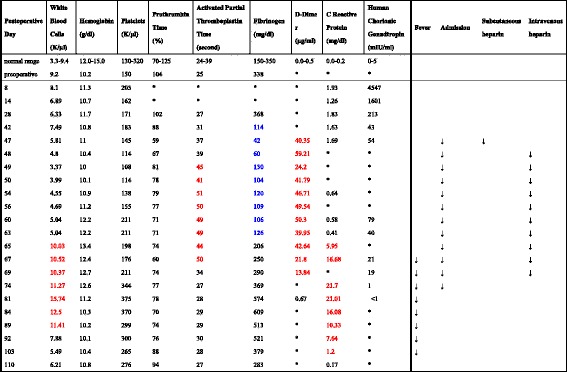
Summary of blood test results

Red font, marked elevated abnormal values; blue font, decreased abnormal values

The asterisk indicates no available data

First, anticoagulant therapy (unfractionated heparin, 10,000 units/day) was administered subcutaneously. However, her serum fibrinogen level did not improve; thus, she received a transfusion of 1200 mL fresh frozen plasma (FFP). However, fibrinogen level did not improve with FFP transfusion. Therefore, activated partial thromboplastin time (APTT)-adjusted intravenous unfractionated heparin (APTT: 1.5- to 2.0-fold increase) was initiated as anticoagulant therapy. Intravenous unfractionated heparin was used from postoperative days 48 to 69, and her serum fibrinogen level successfully improved to 100 mg/dL and heparin-induced thrombocytopenia was not observed (Table 1). Although her serum fibrinogen level was approximately 100 mg/dL for 2 weeks, no symptoms were observed. By postoperative day 65, her serum fibrinogen level markedly improved to 206 mg/dL. However, she had fever (>38 °C), a markedly elevated white blood cell (WBC) count, and an elevated C-reactive protein (CRP) level (Table 1). We initially considered the possibility of an infection; however, a careful physical examination and bimanual palpation of her uterus revealed no sign indicating the presence of infection foci. Blood culture and procalcitonin test results also were negative. We concluded that the fever was induced by absorption of the placenta and not by infection. Therefore, we did not administer antibiotics and treated her with antipyretics instead. Although her fever and markedly increased CRP level persisted, her general condition was good; no clinical signs of infection were observed. On postoperative day 77, she was discharged and careful outpatient observation was continued. T2-weighted MRI on postoperative day 92 revealed a decreased placenta size (approximately 6 cm), and gadolinium-enhanced T1-weighted MRI revealed the lack of gadolinium enhancement of the placenta (Fig. 2c). By postoperative day 103, her fever had resolved. Ultrasonography on postoperative day 121 revealed a normal-sized uterus and no residual placenta. This was confirmed by performing MRI on postoperative day 232 (Fig. 2d). She resumed menstruation 5 months after delivery without any complications.

## Discussion and conclusions

We presented an extremely rare case of placenta percreta complicated with DIC and subsequent fever during conservative management. To reduce maternal complications, placenta accreta has been managed conservatively and 131 of 167 cases (78.4%) have been treated successfully [15]. This management strategy has been associated with a low rate of severe maternal morbidity at centers with adequate equipment and resources. However, it is unknown whether conservative management of placenta percreta has a similar success rate than that of placenta accreta. A previous study reported that despite initial conservative management, 40% of women with placenta percreta subsequently required emergency hysterectomy and 42% experienced major morbidity [13]. Therefore, careful observation is required for conservative management of placenta percreta.

To perform conservative management of placenta percreta safely, it is important to avoid iatrogenic partial separation of the placenta. A transverse uterine fundal incision for the management of placenta accreta can effectively avoid an accidental incision into the placenta and consequently decrease the risk of heavy fetal and maternal hemorrhage [11, 16–18]. Although, in these reports, the total number of cases involving placenta percreta was small, we considered it a useful technique for the conservative management of placenta percreta.

Only a few cases of asymptomatic DIC or fever without infection have been reported during conservative management of placenta percreta. Therefore, we performed a literature search in PubMed/Medline and Google Scholar databases of related English literature published between January 1995 and December 2016 using the keywords “percreta” and “conservative management” or “left” or “placenta in situ.” We excluded reports that lacked detailed descriptions of the patients or with ambiguous reported findings. A total of 68 cases (including our case) reported the detailed conservative management of placenta percreta and conservative management was successful in 40 (58.8%) cases. We read all these articles and selected the cases in which the symptoms were similar (DIC without overt bleeding and infection and maternal fever more than 2 weeks after delivery) to those of our patient [13, 14, 19–27]. Our literature review revealed that 7 of 68 patients (10.3%) had complications of DIC during conservative management of placenta percreta and that 9 of 68 (13.2%) had fever. To date, no study has reported a case as ours wherein both asymptomatic DIC and fever without infection have been successfully treated with medical therapy.

The median time for the onset of DIC was 58 days. A similar cause of DIC was discussed in a previous study. In that report, the patient suffered DIC 49 days after delivery during conservative management of placenta percreta [14]. Emergency hysterectomy was performed, resulting in the rapid normalization of coagulation parameters [14]. In that case, as in ours, it was concluded that the cause of DIC was the residual placenta and the laboratory findings were indicative of fetal death syndrome, which is an extremely rare condition.

To resolve DIC, it is important to eliminate the causative factor. Hysterectomy was performed in five of seven patients, and DIC improved immediately after surgery. However, three of five patients suffered bladder injury and four of five had over 3000 mL of intraoperative bleeding [13, 14, 19–21], even though more than 60 days had elapsed since the cesarean delivery was performed [20]. In our case, as shown in Fig. 2b, the residual placenta invaded the bladder wall; thus, hysterectomy was not performed as it would result in heavy blood loss and severe complications as previously reported. In a previous case report, DIC developed secondary to fetal death syndrome after the death of a single twin in utero [28]. In that case, it was difficult to eliminate the causative factor. However, successful prolongation of this preterm pregnancy with heparin treatment was reported [28]. Therefore, we considered that if a patient had DIC and if performing hysterectomy was difficult, an alternative treatment to avoid hysterectomy would be ideal.

One study reported combined tranexamic acid and enoxaparin use for conservative management of DIC resulting from placenta percreta [22]. Our patient was treated with intravenous APTT-adjusted heparin and could successfully continue conservative management. As in a previous report [22], our case suggested that DIC onset during conservative management of placenta percreta is treated successfully with medical therapy. However, the time needed for DIC resolution may vary; in a previous study [22], DIC resolved within 17 days compared to 22 days in our case. Because our treatment posed the risk of bleeding from the placental attachment site, we considered that our treatment could not be offered as a novel therapy and should be left at the discretion of well-informed patients. Our patient was well informed and strongly desired to continue conservative management. We intravenously administered heparin so as to be able to stop heparin administration and immediately neutralize its effect using protamine sulfate in case of hemorrhage.

Although it is only speculation, the interesting findings of our case revealed that DIC improved at the time of decreased placental blood flow, which was determined by serial MRI. Serial MRI revealed that the blood flow disappeared between day 48 and 92, and DIC improved on day 69. Following decreased placental blood flow, there was a possibility of improvement in DIC like fetal death syndrome during the conservative management of placenta percreta.

Previous cases and our case revealed that DIC without hemorrhage and infection during conservative management of placenta percreta could be medically treated, and continuing the conservative management could avoid the severe complication of hysterectomy. However, prolongation of DIC might place the patient at high risk for complications, such as major bleeding, and the frequency of complications of this treatment is not well understood. If the clinician encounters a similar case, management should be well examined and discussed with the patient.

Our patient had another important issue. The patient experienced fever for approximately 1 month, and her blood test revealed a markedly elevated WBC count and CRP level. We initially considered the possibility of an infection; however, a careful examination, blood culture, and negative procalcitonin test indicated otherwise. The fever subsided with a decrease in the size of the residual placenta. Therefore, we determined that these symptoms were caused by absorption of the placenta. As discussed earlier, placental blood flow disappeared, and the placenta could be necrotic. The inflammatory response to infection and tissue injury supports host defense, clearance of necrotic tissue, adaptation, repair, and absorption of hematoma caused the WBC and CRP elevation [29, 30]. Hence, we speculated that the elevation of WBC and CRP was induced by the absorption of necrotic placenta such as the absorption of hematoma or the clearance of necrotic tissue in our patient. However, it is still difficult to discuss why the fever is limited to observed in the part of patients.

According to the literature review, the presence of fever was considered with infection in all cases except ours. Therefore, the frequency of fever without infection during conservative management of placenta percreta remains unknown. The median time to the onset of fever was 6 weeks after delivery. Our literature review revealed 30 cases of conservative management of placenta percreta that involved the use of antibiotics and 10 cases that did not receive postoperative prophylactic antibiotics. Because we found no incidence of endometritis within 1 week after cesarean delivery in prophylactic antibiotics cases, we considered prophylactic antibiotics to be nonessential [6, 26, 31–33].

Previous studies have reported that procalcitonin is a helpful biomarker for the early diagnosis of sepsis in critically ill patients. Nevertheless, procalcitonin test results must be interpreted carefully in the context of each patient’s medical history, physical examination results, and microbiological assessment [34]. Although it is difficult to rule out infection during conservative management, we considered that procalcitonin was a helpful biomarker to rule out the presence of infection. If the infection can be ruled out, we considered that conservative management with a careful examination can be successful.

Our case is extremely rare; it described the successful management of asymptomatic DIC and subsequent fever after the improvement of DIC during conservative management of placenta percreta. This case illustrated two important clinical experiences. First, DIC secondary to a residual placenta without the hemorrhage and an associated infection can be treated with anticoagulant therapy. This treatment can result in avoidance of hysterectomy, which often results in serious complications. Second, fever and markedly elevated WBC and CRP levels may have been induced by absorption of the residual placenta. A careful examination is needed in such a setting; however, our findings suggested that some patients with DIC and fever can continue conservative management of placenta percreta. Although we described a single case and even though not all patients can be treated in the same way, a very important clinical experience was gained. Further studies are expected to demonstrate the safety and efficacy of conservative management of placenta percreta.

## Abbreviations

APTT: Activated partial thromboplastin time
CRP: C-reactive protein
DIC: Disseminated intravascular coagulation
MRI: Magnetic resonance imaging
WBC: White blood cell
